# Using household survey data to identify large-scale food security patterns across Uganda

**DOI:** 10.1371/journal.pone.0208714

**Published:** 2018-12-13

**Authors:** Jannike Wichern, Joost van Heerwaarden, Sytze de Bruin, Katrien Descheemaeker, Piet J. A. van Asten, Ken E. Giller, Mark T. van Wijk

**Affiliations:** 1 Plant Production Systems, Wageningen University & Research, Wageningen, The Netherlands; 2 Laboratory of Geo-information Science and Remote Sensing, Wageningen University & Research, Wageningen, The Netherlands; 3 International Institute of Tropical Agriculture, IITA-Uganda, Kampala, Uganda; 4 International Livestock Research Institute, Sustainable Livestock Systems, Nairobi, Kenya; Bangor University, UNITED KINGDOM

## Abstract

To target food security interventions for smallholder households, decision makers need large-scale information, such as maps on poverty, food security and key livelihood activities. Such information is often based on expert knowledge or aggregated data, despite the fact that food security and poverty are driven largely by processes at the household level. At present, it is unclear if and how household level information can contribute to the spatial prediction of such welfare indicators or to what extent local variability is ignored by current mapping efforts. A combination of geo-referenced household level information with spatially continuous information is an underused approach to quantify local and large-scale variation, while it can provide a direct estimate of the variability of welfare indicators at the most relevant scale. We applied a stepwise regression kriging procedure to translate point information to spatially explicit patterns and create country-wide predictions with associated uncertainty estimates for indicators on food availability and related livelihood activities using household survey data from Uganda. With few exceptions, predictions of the indicators were weak, highlighting the difficulty in capturing variability at larger scale. Household explanatory variables identified little additional variation compared to environmental explanatory variables alone. Spatial predictability was strongest for indicators whose distribution was determined by environmental gradients. In contrast, indicators of crops that were more ubiquitously present across agroecological zones showed large local variation, which often overruled large-scale patterns.

Our procedure adds to existing approaches that often only show large-scale patterns by revealing that local variation in welfare is large. Interventions that aim to target the poor must recognise that diversity in livelihood activities for income generation *within* any given area often overrides the variability of livelihood activities between distant regions in the country.

## Introduction

Smallholder farming is the basis of living for many of the most vulnerable on earth [1] and one of the most common forms of agriculture worldwide [2]. For the majority of the rural households in sub-Saharan Africa, agriculture contributes a substantial part of their livelihood. There is a strong link of smallholder farming with poverty, malnutrition and hunger of the rural population [3], so that targeting interventions on smallholder farming is important for achieving Sustainable Development Goal 2 (Zero Hunger) of the United Nations. Decision makers need large-scale quantitative information, such as displayed in maps, for targeting interventions, assessing vulnerability of rural households to poverty and food insecurity, and for planning emergency relief.

Maps on poverty and food security are often derived from aggregated information or based on expert knowledge. Until recently, expert-based aggregation masks [4] were typically used. Recent more advanced approaches using machine learning to predict the distribution of poverty from satellite imagery [5] have relied on aggregated household data. While often justified, the aggregation of household data potentially hides relevant information and variability at the household level. After all, poverty and food security tend to be locally driven processes with large variation at small scale, for example between nearby households [6].

An alternative approach would be to model variability at the household level directly, using household survey data in combination with spatially explicit environmental and socio-economic data, and to produce maps that allow identifying spatial patterns at this scale. This would provide a direct estimate of the variability and predictability of welfare indicators at the most relevant scale and would allow evaluation of the importance of explanatory variables at the household level. Such a combination of geo-referenced household level information with spatially continuous information [7, 8] is an underused approach to quantify local and large-scale variation and improve targeting of interventions.

In our study we developed and tested a stepwise procedure to translate point information to spatially explicit patterns and create country-wide spatial predictions of welfare indicators using household survey information. We thereby addressed the following questions:

- Can spatial variability in welfare at the household level be reliably modelled using spatially explicit environmental and socio-economic data in combination with household survey data?
- Do household resource variables offer additional explanatory power compared to environmental and socio-economic variables alone?
- Is there remaining spatial information in unexplained variability that can be exploited for generating spatial predictions?

Welfare and particularly food security was represented using the household food availability framework [9], which enables to identify the importance of different livelihood activities and agricultural products to a household’s potential food availability (as approximation to food security). By differentiating rural households in terms of their livelihood activities and resources contributing to household welfare, the approach generates important insights for effective policy making and provides relevant information for vulnerability and risk assessments [1].

Household level data were acquired from the Living Standard Measurement Study–Integrated Survey on Agriculture (LSMS-ISA) of the World Bank, which provides country-wide household survey data in several countries in sub-Saharan Africa containing information on household welfare and smallholder production [10]. We used the LSMS-ISA data from Uganda [11] as an example because of the country’s large variability in agro-ecosystems and related livelihoods. A majority of households in Uganda lives in rural areas and is involved in agriculture production on small farms [12]. Farming systems in Uganda are diverse, ranging from coffee-banana systems in the humid tropical climate of the south-east to agro-pastoral systems in the semi-arid north. Most farming systems combine crop and livestock activities. Livestock and particularly cattle is dominant in Uganda’s ‘cattle corridor’, an area of pastoral rangelands stretching from the Southwest to the Northeast [13, 14]. Temperature in Uganda mostly depends on elevation and show little annual variation with maxima between 25 to 30 ^o^C. Annual rainfall varies from <750 mm in the northeast to >1750 mm at higher elevations and near Lake Victoria. Rainfall distribution is bimodal in Uganda’s south, but gradually changes to unimodal as we move northeast where the dry season becomes more prolonged [15]. The majority of Ugandans live in the western, central and eastern parts of the country which also have the densest road networks and major towns [16, 17]. While the poverty rate of Uganda has declined in the recent past, it is unclear if this benefited all segments of the population [18, 19]. While the poverty rate of Uganda has declined in the recent past, it is unclear in how far the benefits have been equally distributed [18, 19]. The GINI coefficient (an indicator of inequality) of Uganda increased from 41 in 2012 to 42.8 in 2016 and is similar to neighbouring countries (e.g. 40.8, 37.8 and 45.1 for Kenya, Tanzania and Rwanda, respectively) [20]. This indicates that local variability in poverty and food security is similar in Uganda as other countries in East Africa.

## Material and methods

Protocol on: dx.doi.org/10.17504/protocols.io.vb6e2re

We identified a set of household level indicators to determine food availability (as approximation for food security) and related livelihood activities. We used a regression kriging approach with a set of spatially continuous environmental and socio-economic explanatory variables and household resource explanatory variables (herd size, total cultivated land area, household size) to identify large-scale and local variation.

### Data and data preparation

#### Data

**Fig 1:**
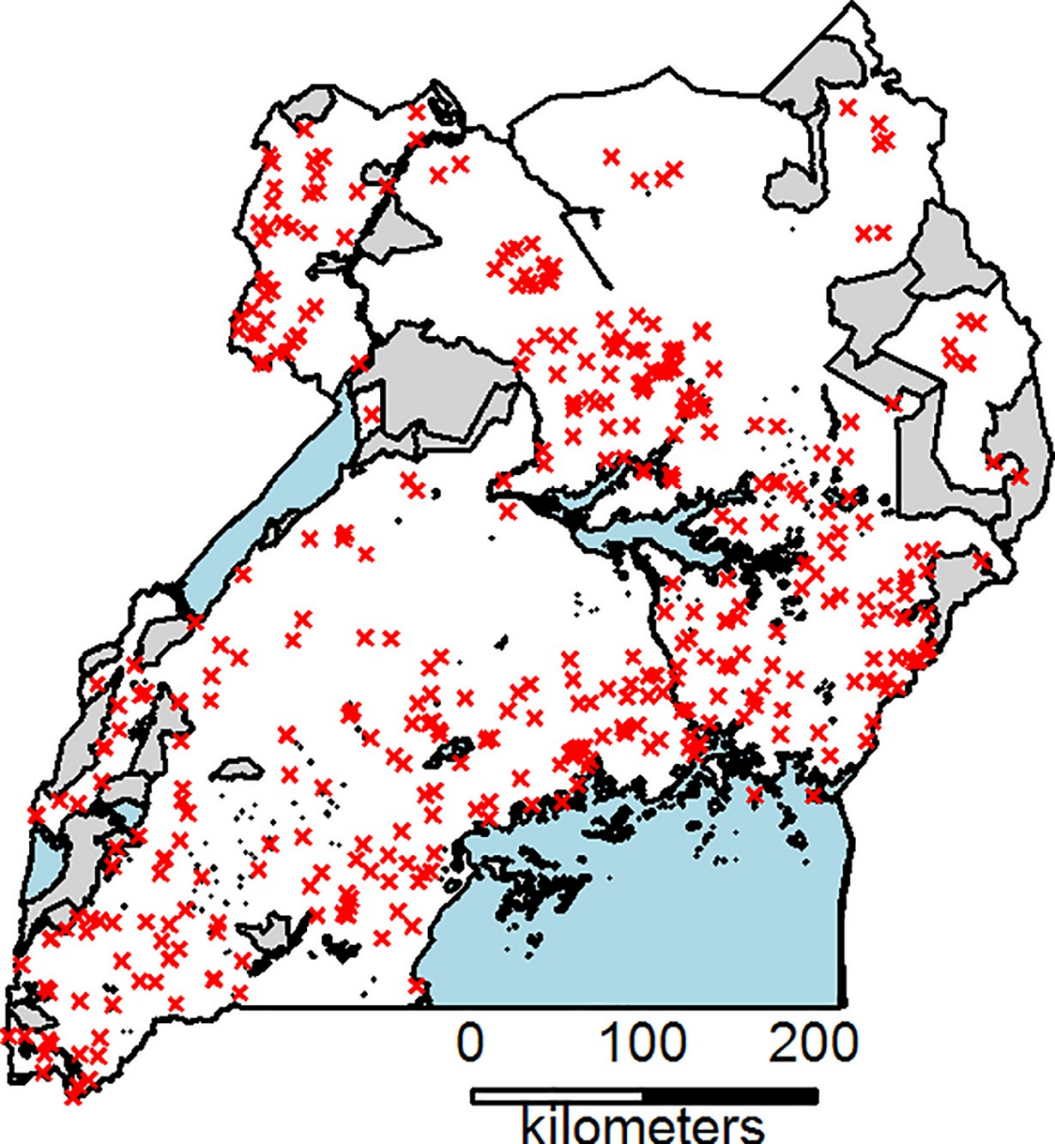
Locations of the households included in analysis (n = 1927). Each red x represents one household. Blue areas are water bodies and grey areas are protected areas (e.g. nature reserves).

We used cross-sectional household survey data for Uganda from the World Bank Living Standard Measurement Study–Integrated Surveys on Agriculture (LSMS-ISA) [10, 11]. In total 2671 geo-referenced households were sampled across Uganda over a 12-month period from 2010 to 2011. The LSMS-ISA has been designed to be nationally representative on rural/ urban and regional levels. Households were sampled per enumeration area (EA), based on a random selection of EAs per region (Kampala, Central, Eastern, Northern and Western) [21]. We used survey data on household characteristics, farm size, crop and livestock production and off-farm income for the household food availability analysis and on herd size, total cultivated land area and household size as household resource explanatory variables for the regression analysis (see below). The three household resource explanatory variables were chosen because they reflect productive resources, which link to the food availability analysis looking at household productivity. We present results from the dataset from the year 2010–11. Other years (LSMS 2011–12) were used to check for consistency of patterns, which revealed that patterns remained similar across years for most variables (for details see S1–S3 Figs). We were interested in the agricultural households and excluded households without land holdings as well as households without any livelihood activities (i.e. no agricultural production and no off-farm income; final sample: 1927 households, Fig 1). For the spatial analysis we collected environmental and socio-economic explanatory variables (henceforth: ‘environmental explanatory variables’) that were available as raster layers, including elevation [22], temperature and rainfall [23], length of growing period [24], soil conditions [25], population density [16] and market access [17] (Table 1).

**Table 1:** Characteristics of the environmental explanatory variables used in the regression analysis.

Code	Spatially continuous environmental variable	Original resolution	Source
DEM	elevation (m.a.s.l.)	3 arc s	Jarvis, Reuter (22)
TEMP	Average annual mean temperature in period 1950–2000 (°C)	30 arc s	Hijmans, Cameron (23)
TEMP_R	Average annual temperature range in period 1950–2000 (max temperature of warmest month minus min temperature of coldest month) (°C)	30 arc s	Hijmans, Cameron (23)
RAIN	Average annual rainfall in period 1950–2000 (mm a^-1^)	30 arc s	Hijmans, Cameron (23)
RAIN_V	Average annual rainfall variation (coefficient of variation calculated on monthly rainfall)	30 arc s	Hijmans, Cameron (23)
LGP	average length of growing period in period 1965–1995 (days/ year)	5 arc min	HarvestChoice (24)
BD	bulk density for three soil layers: 0–5 cm, 5–15 cm, 15–30 cm soil depth (Mg m^-3^)	250 m	Hengl, Mendes de Jesus (25)
SOC	soil organic carbon content for three soil layers: 0–5 cm, 5–15 cm, 15–30 cm soil depth (kg C Mg^-1^)	250 m	Hengl, Mendes de Jesus (25)
SCARB	soil carbon stock, calculated from soil data, mean across three layers (kg C Mg^-1^)	-	-
POP	human population density (number km^-2^)	~100 m	WorldPop (16)
TRAV	market access in travel time to nearest town of +50,000 inhabitants based on road network from 2000 (minutes)	30 arc s	Nelson (17)

#### Data preparation and calculation of food availability indicator

The household locations were randomly displaced by the publishing authority with an offset ≤ 10 kilometres and several households were clustered resulting in multiple households relocated to the same location [11]. For the kriging unique locations are required. We therefore randomly offset the household locations using a random distance of ≤ 50 meters and a random direction. We sampled the values of the raster layers of the environmental explanatory variables at these new household points. To minimize the unknown errors in the sampled environmental explanatory variables introduced from offsetting the household locations, we resampled all raster layers to a resolution of 5 arc minutes, which approximates a grid cell size of 10 x10 km near the equator. Given the small spatial offset compared with the country-wide scale of analysis, we consider the remaining uncertainty to be negligible. Raster layers and household points were geo-referenced to WGS84 coordinates.

We calculated soil carbon stock *SCARB* (kg C Mg^-1^) from the soil organic matter content *SOC* (kg C Mg^-1^) and the bulk density *BD* (Mg m^-3^) for three soil layers of increasing thickness *Δz* (m, 0–0.05 m, 0.05–0.15 m, 0.15–0.3 m):

$$SCARB=BD\times ∆z\times SOC\times 10^{4}$$ [1]

We calculated the mean carbon stock value across the three layers for each grid cell.

To approximate food security we chose the food availability framework by [9] because it enables to identify the importance of different livelihood activities, crops and livestock types for a household’s welfare (in terms of food availability). Although the food availability indicator (FA) does not consider all dimensions of food security [26], it closely correlates with well-established indicators such as the Household Dietary Diversity Index (HDDS) and the Household Food Insecurity Access Scale (HFIAS) [27]. For each household we calculated the FA estimating the potential amount of daily food energy that is available to a male adult equivalent household member (kcal cap^-1^ d^-1^):

$$FA=\frac{{(E}_{consumed}+E_{income})}{365\times n_{hh-mae}}$$ [2]

Where *E*_*consumed*_ is the annual direct consumption of potential food energy from on-farm products (kcal a^-1^), *E*_*income*_ is the annual indirect consumption of potential food energy from on- and off-farm income (kcal a^-1^), and *n*_*hh-mae*_ is the household size in male adult equivalents. *E*_*consumed*_ was derived from information on produced amounts of crops and livestock products and the respective kilo-caloric energy values of the crops and livestock products. These were obtained from the standard product list of the US Department of Agriculture (source: ndb.nal.usda.gov/ndb/search/list, accessed 02/07/16) and from the FAO (source: http://www.fao.org/docrep/x5557e/x5557e00.htm#Contents, accessed 02/07/16). Consumption was then quantified from the difference between produced and sold quantities of the respective product. Total household income was calculated from reported quantities of crop and livestock product sales and from off-farm income. Assuming that all this income could have been used to purchase the staple food (maize) we translated the monetary value to kilocalories equivalent (*E*_*income*_). Household size was standardised to male adult equivalence (*n*_*hh-mae*_) using sex and age dependent daily energy requirements relative to that of an adult male (2500 kcal cap^-1^d^-1^) [28].

Livelihood activities were derived from the different food energy sources contributing to FA and were expressed as relative contribution to FA (values between 0 and 1, Table 2 and S1 Table). We distinguished between livelihood activities on-farm (crop contribution to FA, livestock contribution to FA) and off-farm (off-farm income contribution to FA). We subdivided the on-farm livelihood activities into key crops (highland banana, sorghum, maize, cassava, beans, and coffee) and livestock types (cattle and poultry) that contributed to the crop and the livestock part of the FA, respectively. Details on the methodology are provided in [29]. The data analysis was performed in R (version 3.2.3) [30].

**Table 2:** Food availability indicators as response variables and environmental and household resource explanatory variables of the analyses.

Response variables (food availability indicators)	Spatially continuous environmental explanatory variables	Household resource explanatory variables
Household food availability (FA)	Elevation	Total cultivated land area
*Variables contributing to FA (livelihood activities)*:	Mean annual temperature	Household size
Crop contribution	Annual temperature range	Herd size
Livestock contribution	Annual rainfall	
off-farm income contribution	Annual rainfall variation	
*Variables contributing to the crop part of FA*:	Length of growing period	
banana contribution	Soil carbon stock	
sorghum contribution	Human population density	
maize contribution	Market access	
cassava contribution		
beans contribution		
coffee contribution		
*Variables contributing to the livestock part of FA*:		
cattle contribution		
poultry contribution		

### General approach

**Fig 2:**
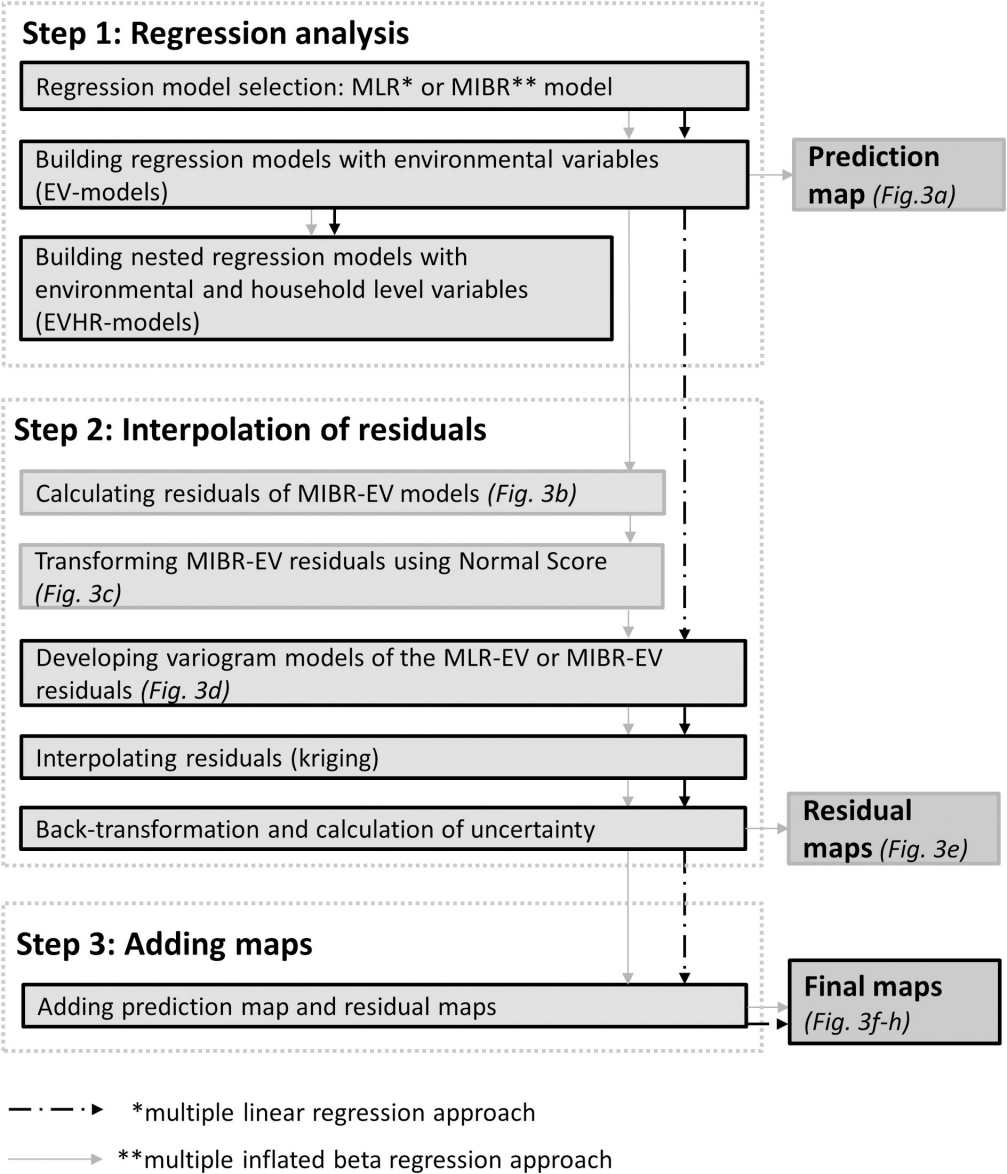
The stepwise procedure. See text for further explanation.

**Fig 3:**
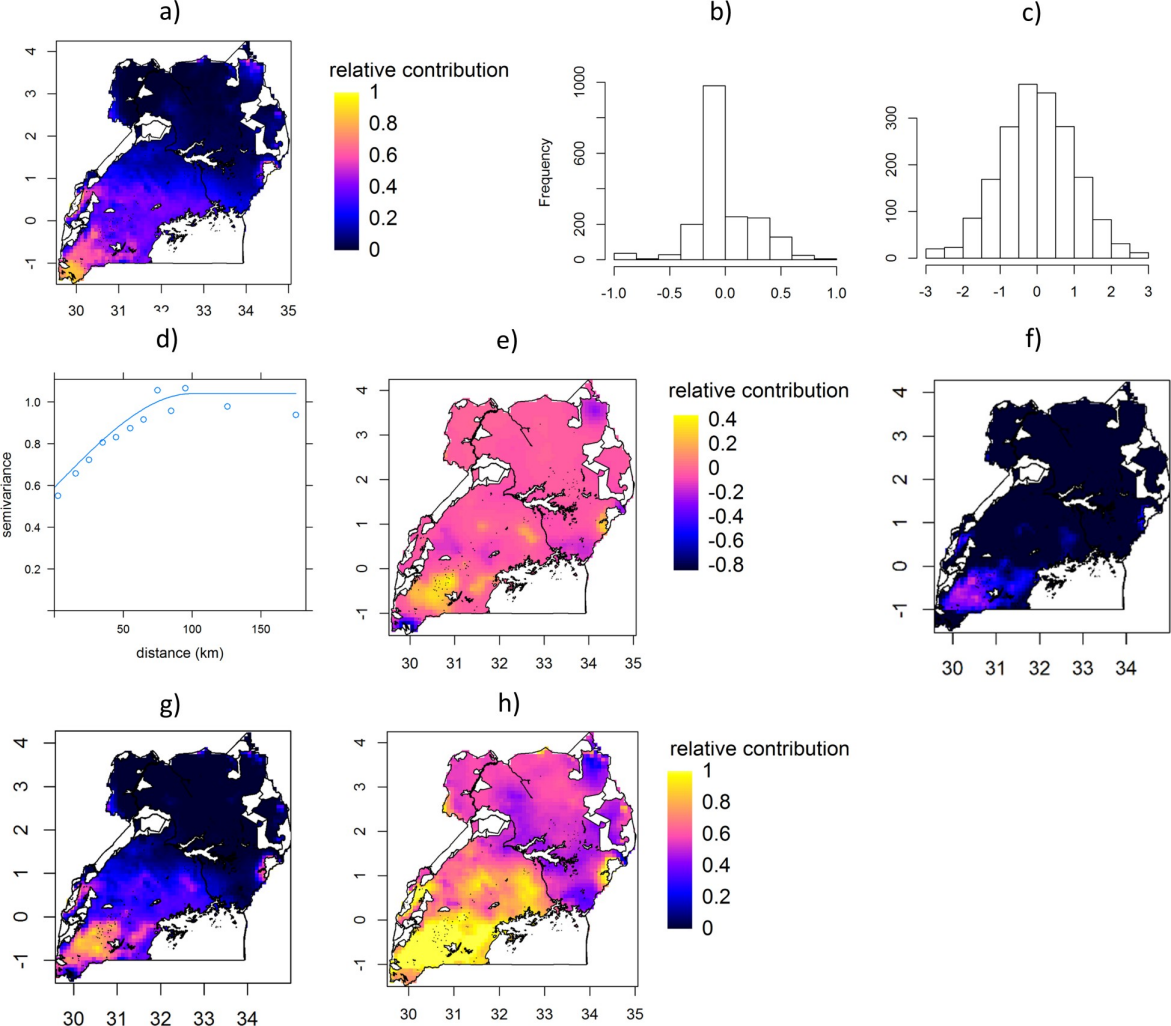
Mapping livelihood activities contributing to food availability (FA) following the procedure of Fig 2 on the example of highland banana contribution to the crop part of FA. a) Highland banana contribution predicted by a multiple inflated beta regression (MIBR) model. b) Histogram of the regression residuals from the MIBR model. c) Histogram of the regression residuals after Normal Score Transformation. d) Variogram model of the transformed regression residuals. e) Unexplained structure in the regression residuals interpolated by kriging, after back-transformation. f) Prediction map (a) added to the lower 95% bound of the prediction interval (PI) of the interpolated residuals. For *y* < 0, values are set to 0. g) Prediction map (a) added to the interpolated residuals (e). For *y* > 1, values are set to 1; for *y* < 0, values are set to 0. h) Prediction map (a) added to the upper 95% PI of the interpolated residuals. For *y* > 1, values are set to 1. White areas are protected areas (e.g. national parks) and water bodies.

Our procedure aims at generating a spatial prediction of food availability indicators using household and environmental data, taking full account of variability at the household level. It combines a predictive model of household level variation for an indicator of interest with a spatial model of unexplained variation. Although machine learning algorithms have been used successfully for building predictive models of poverty indicators, we opted for a regression approach to allow for formal tests of model improvement when using different types of explanatory variables. By comparing predictive models using both environmental and household resource explanatory variables to models only using environmental explanatory variables we established to what extent model fit from environmental data was sufficient to perform prediction on a spatial grid. Modelling of the unexplained variation (i.e. the model residuals) was done by a classic spatial kriging approach (see below). Finally spatial predictions based on explained and unexplained variation were combined into a single map. A detailed description of the procedure (Fig 2) is provided in the following sections. An example of the procedure for highland banana contribution is illustrated in Fig 3 (Results).

### Step 1: Regression analysis

#### Regression model selection

The indicators FA and the contributions of crops, livestock and livelihood activities to FA were the response variables in the regression models and the environmental variables were the explanatory variables (Table 2). If the indicator was transformable so that regression residuals were approximately normally distributed, a multiple linear regression (MLR) model was used. This was possible for FA after log-transformation. The contributing livelihood activities, variables representing proportions containing a large number of zeros and ones, were not transformable to a (near-)normal distribution and required an alternative regression model.

For this purpose we used a multiple zero-and-one inflated beta regression (MIBR) model that can handle distributions where the observation, *y*, is a proportion including many zeros and ones [31, 32]. The MIBR model uses a mixed continuous-discrete distribution that defines both the probabilities P_0_ (*y* = 0), P_1_ (*y* = 1) and 1- P_0_- P_1_ (0 < *y* < 1) and the probability distribution of the values of y between 0 and 1, which is given by a beta distribution with shape parameters α and β. This complete probability distribution of *y* is defined by four parameters, *μ*, *σ*, *ν* and *τ*, where *μ*, *σ* define the shape of the beta distribution as α = *μ(1- σ*^*2*^*)/ σ*^*2*^, β = (1- *μ)(1- σ*^*2*^*)/ σ*^*2*^ while *ν* and *τ* describe the probabilities P_0_ = *ν*/(1+*ν* + *τ)* and P_1_ = *τ* /(1+*ν* + *τ)*. The expectation of *y* under this distribution is given by E(*y*) = (*τ + μ)/(*1+*ν* + *τ)* [33]. For variables with no or few (i.e. ≤ 3) observations of *y* = 1, we considered the sample too small for making reasonable predictions of *y* = 1. Therefore, we used a multiple zero-inflated beta regression model (MIBR-0), leaving out the distribution parameter *τ*. This was the case for the contributing livelihood activities ‘coffee’ and ‘livestock’.

MLR models were estimated using the ordinary least squared method and the R package ‘stats’. The MLR model fit was evaluated based on R-Squared (*R*^*2*^). MIBR were fitted by maximum-likelihood methods using the R package ‘gamlss’ [34]. Details are in [35]. The MIBR model fit was evaluated by the squared correlation of fitted and observed *y* (henceforth ‘pseudo R^2^’).

#### Building regression models with environmental explanatory variables (EV-models)

For each indicator, the environmental explanatory variables that were used to build the regression models were selected in two steps: First, we built simple MLR or MIBR models for each environmental explanatory variable and selected those variables that gave a significant slope in the simple models at *p* < 0.1 for further analysis. Second, we analysed the significant environmental explanatory variables on collinearity using the variance inflation factor (*VIF*) approach (R package ‘usdm’) and included all environmental explanatory variables with *VIF* < 10 in the multiple regression analysis [36].

The MLR model was fit using the step-function with forward direction. The MIBR model was fit using the stepGAICAll.A function (R package ‘gamlss’), which uses a forward selection and backward elimination approach for each model parameter and selects the best final model based on the AIC. To reduce the chance for model overfitting, we simplified the selected best MIBR model (full model) by removing the last coefficient of any of the model parameters (μ, σ, ν and τ) if that one had a significance of p < 0.1. We rejected the simplified model if the difference between the AIC of the full model and the AIC of the simplified model > 10 [37]. The regression models were used to generate prediction maps for each indicator for the entire country. For the MIBR models we calculated the predicted y-value ‘*y*_*predicted*_’ [35] to generate prediction maps.

#### Building nested regression models with environmental and household resource explanatory variables (EVHR-models)

To identify how much of the variation was explained by the environmental explanatory variables in comparison with the household level variables, we built nested regression models for each indicator: We used the regression model with environmental explanatory variables (EV-model, see step 2) and added household resource variables (EVHR-model). The household resource variables had first been tested on collinearity using the VIF approach and no collinearity was observed.

### Step 2: Spatial interpolation of regression residuals

The regression models did not capture all the spatial structure in the data. To account for remaining spatial structure, we interpolated the residuals of the MLR and MIBR models.

#### Calculation and transformation of MIBR residuals

While the residuals of the MLR model could be directly derived from the MLR model results, the MIBR residuals were calculated at the household locations:

$${Residuals}_{MIBR,i}=y_{observed,i}-y_{predicted,i}$$ [3]

Where *y*_*observed*_ is the value of the indicator *y* observed for household *i* and *y*_*predicted*_ is the value of the indicator *y* predicted from the MIBR model at the location of household *i*. To account for the spatial interpolation requirement of normally distributed residuals, the MIBR regression residuals were transformed using a quantile-based normal score transformation for 100 intervals [38]. With this approach the tails of the distribution (< 0.5% and > 99.5%) were truncated. This trade-off was acceptable, since we were interested in the larger patterns rather than in predictions for particular regions.

#### Variogram models of the MLR and MIBR residuals

To identify spatial autocorrelation in the residuals, we fitted variogram models using the method of moments with weights based on the number of point pairs over the squared distance. We selected the best variogram models based on the weighted sum of squared errors and verified the variogram models by cross-validation (krige.cv-function of the R package ‘gstat’). Variogram models are described by three main characteristics, the nugget, the sill and the range. The nugget is the value at which the variogram (almost) crosses the y-axis and indicates the level of local variation at distances smaller than the sampling interval. The sill is the y-value at which the curve of the variogram model flattens out indicating the level of spatial autocorrelation. The range indicates the distance at which the curve of the variogram model flattens out and thus the distance of spatial autocorrelation (https://gisgeography.com/semi-variogram-nugget-range-sill/, accessed 16/07/18). We calculated the nugget-sill ratio to identify how much of the variation in the residuals was explained by spatial autocorrelation in relation to local variation.

#### Interpolation of the MLR and MIBR residuals

For the MLR model, we used the residual variogram model in combination with regression kriging (R package ‘gstat’) to compute a final map of the log-transformed FA. This approach takes into account spatial correlations of the regression residuals in the regression model fit. For the MIBR models we used the variogram models of the normal score transformed residuals to interpolate the transformed residuals by kriging and produce residual maps. We used the MIBR model as a deterministic surface, which was a model choice implying that all uncertainty was attributed to lack of fit of the MIBR models in the further steps of the procedure. Subsequently, all uncertainty was captured in the prediction intervals of the interpolated MIBR residuals (see below).

#### Back-transformation and calculation of uncertainty

For the MLR model, the kriging results of the log-transformed FA were back-transformed to obtain the expected median *E(Y)* of FA [39]:

$$E(Y)=e^{\mu }$$ [4]

Where *μ* is the mean of the log-transformed FA. The uncertainty of the expected mean of FA was identified calculating the back-transformed upper and lower 95% bounds of the prediction interval (*PI*_*95*, *u*, *MLR*_; *PI*_*95*, *l*, *MLR*_):

$${PI}_{95,u/l,MLR}=e^{\left(\mu \pm 1.96\times \sigma \right)}$$ [5]

Where *σ* is the standard deviation of the log-transformed FA. For the MIBR model, the upper and lower 95% bounds (*PI*_*95*, *u*, *MIBR*_; *PI*_*95*, *l*, *MIBR*_) of the interpolated transformed prediction intervals were calculated as follows:

$${PI}_{95,u/l,MIBR}=\mu \pm 1.96\times \sigma$$ [6]

Where μ is the mean and σ the standard deviation of the interpolated transformed residuals. The interpolated means and upper and lower 95% bounds of the prediction interval of the transformed MIBR residuals were back-transformed using the inverse of the normal score transformation. In this way, the mean of the transformed residuals represents the median of the back-transformed residuals.

### Step 3: Adding the prediction and residual maps

For the MLR models step 2 and 3 were performed in one step using regression kriging (R package ‘gstat’). For the MIBR models the prediction map and the back-transformed median residual map were added to generate a final map of the variable. Uncertainty in the interpolated MIBR residuals was mapped by adding the MIBR prediction map and the back-transformed MIBR residual maps of the upper and lower bounds of the prediction interval, respectively. Where *y* > 1 or *y* < 0 as a result of the addition, values were corrected to remain within the bounds of proportional data (0,1).

## Results

### The stepwise procedure

The stepwise procedure is illustrated with the example of highland banana contribution to the crop part of FA (Fig 3). Highland banana contribution was first predicted based on the MIBR model (step 1, Fig 3A). The regression residuals (Fig 3B) were transformed to a normal distribution using normal score (Fig 3C) and spatial autocorrelation of the transformed residuals was quantified in the variogram model (step 2, Fig 3D). The transformed residuals were interpolated using the variogram model and simple kriging and were then back-transformed to generate a map of the median residuals (Fig 3E). The prediction map was added to the back-transformed median of the interpolated residuals, lower 95% bound of the prediction interval (PI) and upper 95% PI of the interpolated residuals (step 3, Fig 3F–3H). Results of the single steps for all indicators are discussed below.

### Regression models using environmental explanatory variables

Spatial variation in FA was explained by the length of growing period, soil carbon stock, annual rainfall variation, population density and travel time to the market, but explanatory power was low (R^2^ = 0.10, Table 3). Among the MIBR-EV models, the models predicting highland banana and sorghum contributions revealed the largest explanatory power (R^2^ = 0.35 and 0.53, respectively), while for all other indicators the model performance was poor (R^2^ ≤ 0.12, Table 4, details S2–S4 Tables). Variation in highland banana contribution was explained by annual mean temperature, annual temperature range, annual rainfall, soil carbon stock and travel time to the market. Variation in sorghum contribution was explained by annual temperature range, annual rainfall variation, length of growing period and soil carbon stock. The results indicate that the available environmental explanatory variables explained little of the variation for most of the indicators (except for highland banana and sorghum).

**Table 3:** Multiple linear regression (MLR) model of food availability (FA) with environmental explanatory variables (MLR-EV) and with environmental and household resource explanatory variables (MLR-EVHR) as explanatory variables.

Explanatory variable	Coefficient	Beta^a^	Std. Error	t-Statistic	Sign. (p-value)
***MLR-EV model***					
(intercept)	7.34	-	5.99 x 10^−1^	12.25	***
LGP	6.75 x 10^−3^	0.12	1.57 x 10^−3^	4.29	***
SCARB	1.94 x 10^−5^	0.16	3.49 x 10^−6^	5.55	***
RAIN_V	-2.13 x 10^−2^	-0.11	5.37 x 10^−3^	-3.96	***
POP	-2.23 x 10^−2^	-0.10	5.35 x 10^−3^	-4.16	***
TRAV	-1.13 x 10^−3^	-0.08	3.60 x 10^−4^	-3.15	**
R-squared	0.10				
Adjusted R-squared	0.10				
F-statistic	42.27				
***MLR-EVHR model***					
(intercept)	7.62	-	5.93 x 10^−1^	12.84	***
LGP	6.20 x 10^−3^	0.11	1.55 x 10^−3^	3.99	***
TLU	4.31 x 10^−2^	0.16	5.94 x 10^−3^	7.24	***
SCARB	2.03 x 10^−5^	0.16	3.45 x 10^−6^	5.90	***
RAIN_V	-2.21 x 10^−2^	-0.11	5.29 x 10^−3^	-4.18	***
POP	-2.00 x 10^−2^	-0.09	5.28 x 10^−3^	-3.78	***
TRAV	-1.32 x 10^−3^	-0.10	3.56 x 10^−4^	3.72	***
HH_SIZE	-2.28 x 10^−2^	-0.06	8.25 x 10^−3^	-2.76	**
LAND	8.78 x 10^−3^	0.04	4.44 x 10^−3^	1.98	*
R-squared	0.13				
Adjusted R-squared	0.13				
F-statistic	35.31				

Explanatory variables: LGP = average length of growing period, RAIN_V = average annual rainfall variation, RAIN = average annual rainfall, SCARB = mean soil carbon stock, TRAV = market access in travel time to nearest town of +50,000 inhabitants, LAND = total cultivated land area, TLU = herd size, HH_SIZE = number of household members

Significance

*** < 0.001

** < 0.01

* < 0.05,. < 0.1

^**a**^Beta = coefficient X standard deviation of variable x / standard deviation of FA

**Table 4:** Regression results of the multiple inflated beta regression model with environmental explanatory variables (MIBR-EV) and with environmental and household resource variables (MIBR-EVHR) as explanatory variables.

	Indicator	Crop contribution	Livestock contribution	Off-farm income contribution	Banana contribution	Sorghum contribution	Maize contribution	Cassava contribution	Coffee contribution	Beans contribution	Cattle contribution	Poultry contribution
***MIBR-EV model***												
	**Pseudo R**^**2**^	**0.02**	**0.003**	**0.01**	**0.35**	**0.53**	**0.02**	**0.12**	**0.05**	**0.04**	**0.005**	**0.01**
	AIC	-7397	1774	2350	1686	1164	1362	877	1295	-113	2303	1727
	AICini—AIC_final_	77	47	89	1052	530	141	573	197	390	24	40
	*Explanatory variables*:											
	DEM			μ, σ								
	TEMP				μ, ν		μ, σ	μ, σ, ν, τ	μ, σ, ν	ν		
	TEMP_R			μ, σ	μ, ν	μ, ν	ν, τ	ν				
	RAIN				μ, ν			μ, σ, ν				
	RAIN_V	μ, ν				μ, ν					τ	σ, ν
	LGP	μ, σ	μ, σ	μ, ν, τ		μ, σ, ν, τ	μ, σ, ν, τ	ν	μ, ν			
	SCARB				μ, ν	τ	ν, τ			μ, ν		
	POP		μ, ν	μ								
	TRAV	τ	μ, ν	μ, σ	ν			ν	ν		σ, ν	ν
***MIBR-EVHR model***					*not converging*							
	**Pseudo R**^**2**^	**0.05**	**0.17**	**0.02**		**0.54**	**0.02**	**0.12**	**0.06**	**0.05**	**0.29**	**0.007**
	AIC	-7611	1378	2305		1156	1344	865	1279	-128	1785	1704
	AICini—AIC_final_	291	443	134		538	159	585	212	405	535	63
	*Explanatory variables*:											
	DEM			μ, σ								
	TEMP						μ, σ,	μ, σ, ν, τ	μ, σ, ν	ν		
	TEMP_R			μ, σ		μ, ν	τ	ν				
	RAIN							μ, σ, ν				
	RAIN_V	μ, σ, ν				μ, ν					τ	σ, ν
	LGP	μ, σ	μ, σ	μ, ν, τ		μ, σ, ν, τ	σ, ν, τ	ν	μ, ν			
	SCARB					τ	ν, τ			μ, ν		
	POP		μ, ν	μ, σ								
	TRAV	τ	ν	μ, σ				ν	ν		σ, ν	ν
	TLU	μ, σ, ν, τ	μ, σ, ν	μ, σ		σ, τ			σ, ν		ν	ν, τ
	HH_SIZE	μ, σ, τ	ν				μ, σ, ν	μ	ν	μ, σ	ν, τ	
	LAND	μ, τ		μ, σ				ν				

For explanation of model parameter see Material and Methods. Explanatory variables: DEM = elevation, TEMP = average annual mean temperature, TEMP_R = average annual temperature range, RAIN = average annual rainfall, RAIN_V = average annual rainfall variation, LGP = average length of growing period, SCARB = mean soil carbon stock, POP = human population density, TRAV = market access in travel time to nearest town of +50,000 inhabitants, LAND = total cultivated land area, TLU = herd size, HH_SIZE = number of household members. Explanatory variables explain model parameters at significance p < 0.1

### Nested regression models

Adding the household resource explanatory variables herd size, total cultivated land area and household size to the MLR-EV model (MLR-EVHR) increased the explanatory power for FA somewhat (R^2^ = 0.13, against 0.1 for the MLR-EV model, Table 3), but most of the variation remained unexplained. Our results differed from findings of [9], who explained a larger part of FA with herd size, total cultivated land area and household size (R^2^ = 0.33 using artificial neural networks). Adding household resource explanatory variables to the MIBR-EV models (MIBR-EVHR) raised explanatory power only for the livestock and cattle contribution models due to the strong link between livestock and cattle contribution and herd size (pseudo R^2^ = 0.17 and 0.29, against 0.003 and 0.005 for the MLR-EV model; Table 4, details S5–S7 Tables). Explanatory power did not increase for the models of crop contribution.

### Spatial structure in the regression residuals

We chose a nugget-sill ratio ≤0.7 to indicate spatial autocorrelation. Seven indicators (highland banana, sorghum, maize, cassava and coffee contributions to the crop part of FA, and cattle and poultry contributions to the livestock part of FA) revealed spatial autocorrelation in the regression residuals with varying strength. While spatial autocorrelation accounted for 52% of the variance in the regression residuals of coffee contribution, this was only 30% for cassava (nugget-sill ratios 0.48 and 0.7, respectively). The other variables (FA, livestock and off-farm income contributions to FA and beans contribution to the crop part of FA) had little or no spatial autocorrelation in their regression residuals. For crop contribution to FA the variogram model did not converge, but the observed (experimental) variogram indicates little spatial autocorrelation (Table 5, S4 Fig). Particularly for FA and off-farm income contribution the curves of the variogram models were flat (small sill) and nuggets were large. The range of spatial autocorrelation of the regression residuals of the seven indicators with nugget-sill ratio ≤ 0.7 varied from 40 km for the residuals of coffee contribution to 175 km for the residuals of sorghum contribution.

**Table 5:** Parameter of the variogram models of the transformed regression residuals for each indicator.

Indicator	FA	Crop contribution	Livestock contribution	Off-farm income contribution	Banana contribution	Sorghum contribution	Maize contribution	Cassava contribution	Coffee contribution	Beans contribution	Cattle contribution	Poultry contribution
*Variogram model*												
Model shape	matern*	*Model not converging*	matern*	matern*	spherical	spherical	spherical	matern*	matern*	matern*	matern*	matern*
Nugget	1.32		0.76	0.81	0.53	0.52	0.67	0.72	0.50	0.63	0.55	0.62
Sill	1.61		0.98	0.91	0.98	0.91	1.04	1.02	1.06	0.88	1.03	1.06
Range (km)	87		27	70	87	175	124	43	40	19	43	57
**Nugget-sill ratio**	0.82		0.78	0.89	0.54	0.57	0.64	0.70	0.48	0.72	0.54	0.59
(1- nugget-sill ratio)*100 (%)	18		22	11	46	43	36	30	52	28	46	41

lag boundaries at 10, 20, 30, 40, 50, 60, 70, 80, 90, 100, 150, 200; cut-off at 200 km

* kappa = 0.5

No fit for crop contribution

### Mapping FA and contributing livelihood activities

**Fig 4:**
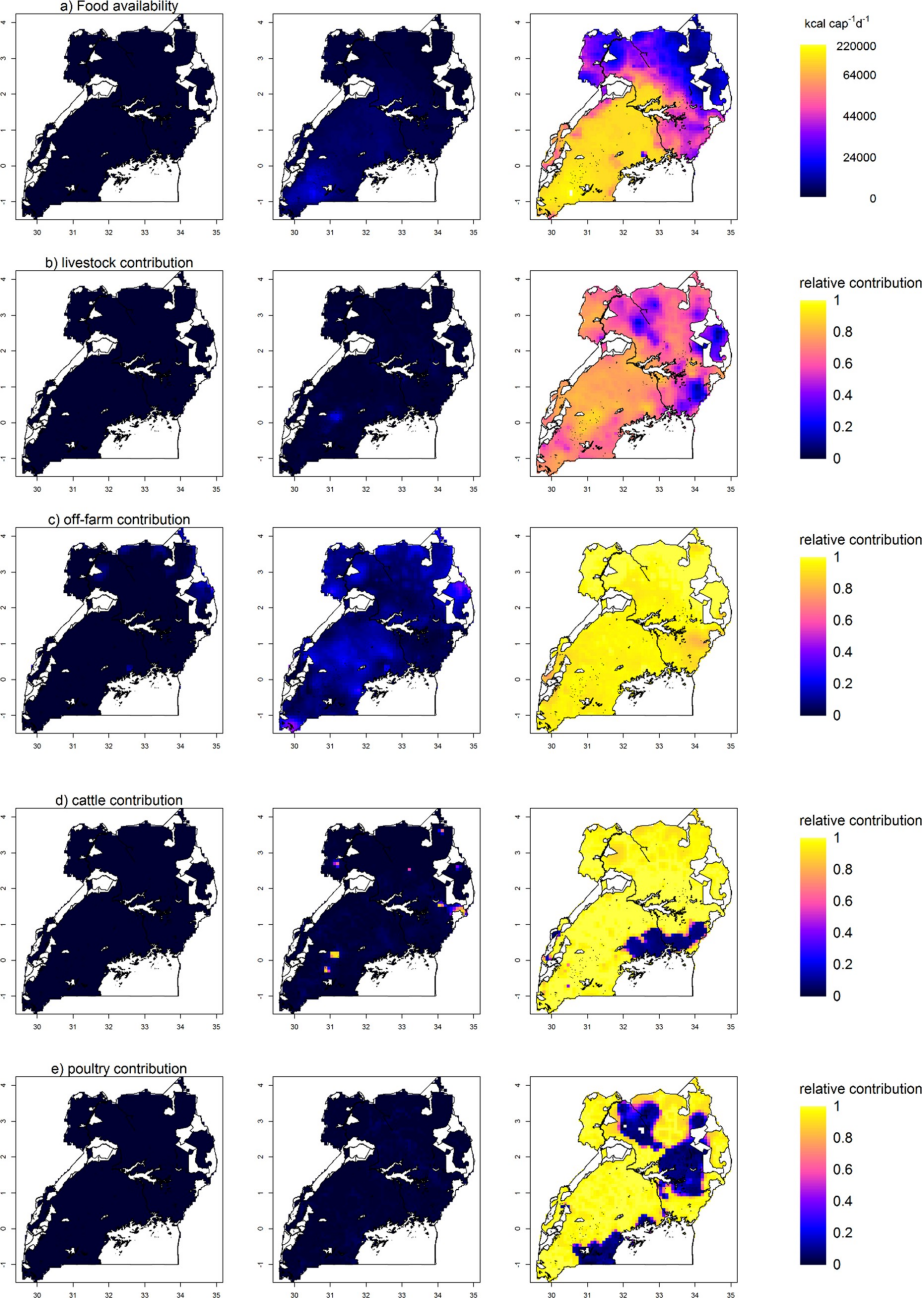
Predictions and uncertainty maps of the food availability indicators FA, off-farm income, livestock, cattle and poultry contributions. a) Predicted food availability (FA, in kcal cap^-1^ d^-1^) across Uganda. Left: Lower 95% bound of prediction interval (PI); Middle: median FA; Right: upper 95% PI. b-e) Livelihood activities contributing to FA across Uganda. Left: Lower 95% PI (MIBR prediction map + lower 95% PI of residuals; for *y* < 0, values are set to 0); Middle: MIBR prediction map + median residuals; for y>1, values are set to 1; for y<0, values are set to 0); Right: Upper 95% PI (MIBR prediction map + upper 95% PI of residuals; for *y* > 1, values are set to 1). White areas are protected areas (e.g. national parks) and water bodies.

**Fig 5:**
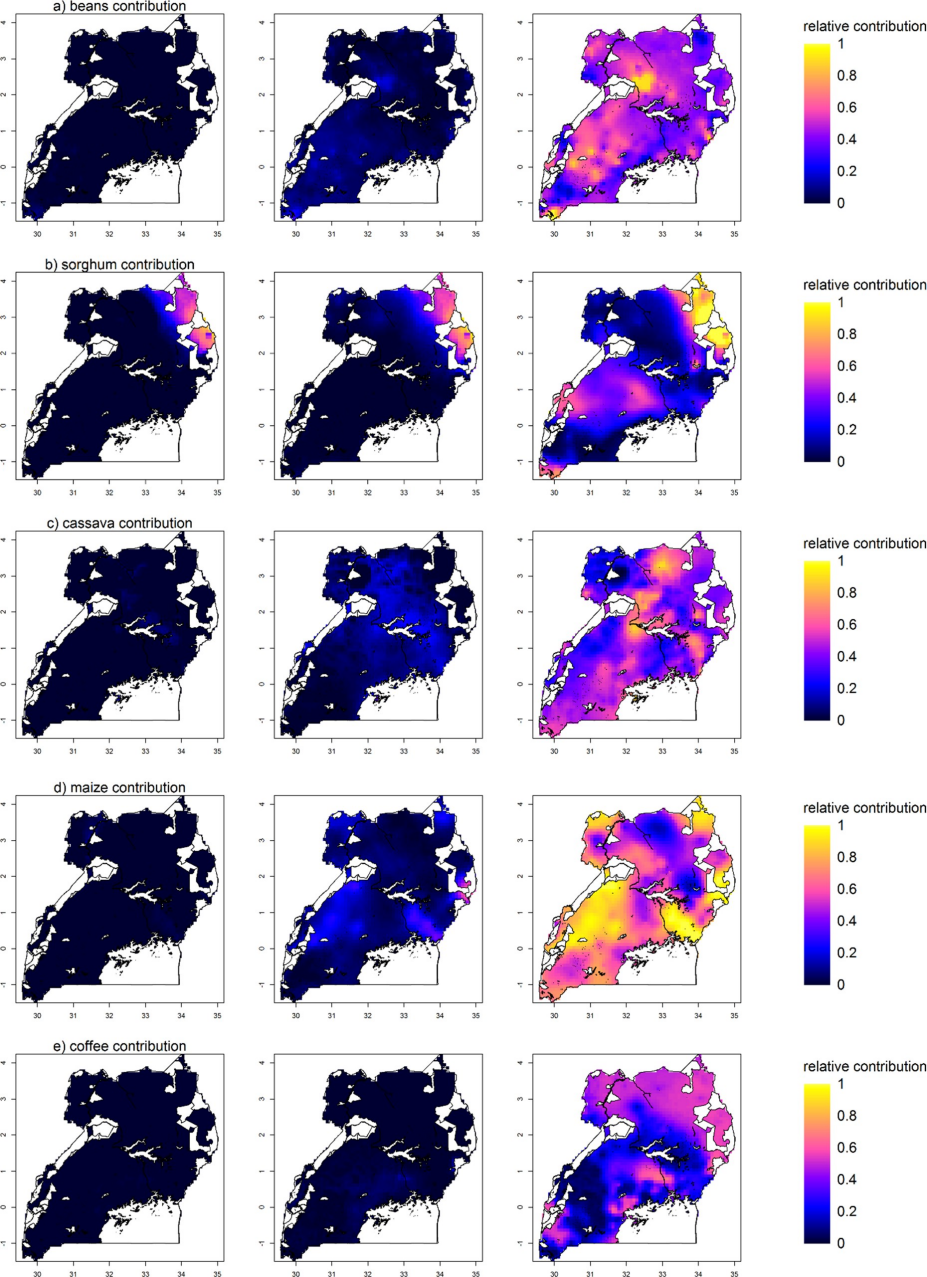
Predictions and uncertainty maps of the food availability indicators beans, sorghum, cassava, maize and coffee contributions. Left: Lower 95% PI (MIBR prediction map + lower 95% PI of residuals; for *y* < 0, values are set to 0); Middle: MIBR prediction map + median residuals; for y>1, values are set to 1; for y<0, values are set to 0); Right: Upper 95% PI (MIBR prediction map + upper 95% PI of residuals; for *y* > 1, values are set to 1). White areas are protected areas (e.g. national parks) and water bodies.

Application of the stepwise procedure to FA identified a larger FA in the southwest of Uganda (median values ≥ 15,000 kcal cap^-1^ d^-1^) and the smallest FA in the north and northeast (median values < 5000 kcal cap^-1^ d^-1^) (Fig 4A middle). However, the large local variation of the regression residuals indicated much spatial uncertainty about FA (Fig 4A left and right). The maps of the contributions of livestock, off-farm income and beans indicated uniform patterns across the country with few hotspots for livestock and off-farm income contribution (Fig 4B and 4C, Fig 5A middle). Also here the uncertainty in the patterns was large (Fig 4B and 4C, Fig 5A left and right) due to an enormous local variation in the regression residuals (nugget-sill ratios ≥ 0.72) and poor explanatory power of the MIBR-EV models (pseudo R^2^ ≤ 0.04). These results indicate that spatial variation was huge at a range shorter than supported by the resolution of the environmental explanatory variables (<10 km).

In contrast, the highland banana and sorghum contributions revealed distinct large-scale spatial patterns. Highland banana contribution was largest in Uganda’s Southwest, around Lake Victoria, in the highland regions and in central Uganda (Fig 3G). Uncertainty in these patterns was large in the central areas and in the mountain ranges (Fig 3F and 3H) as a result of large local variation in the residuals (Fig 3D and 3E) and potentially also due to low observation density at some locations. Sorghum contribution was largest in the Northeast of Uganda (Fig 5B). While uncertainty in the residuals of sorghum contribution was low for most of the country, the upper 95% bound of the prediction interval of the residuals indicated that sorghum contribution might be under-predicted in the Northeast, parts of the central area and of the west and southwest of the country. The MIBR-EV models of highland banana and sorghum contributions were the only models that had a pseudo R^2^ > 0.3, resulting in smaller residuals than for the other variables. Cassava, maize and coffee contributions indicated weak large-scale patterns (Fig 5C–5E), and uncertainty in these patterns was huge, attributed to the large local variation of regression residuals indicated in the variogram models (nugget-sill ratios ≥ 0.64, exception coffee: 0.48) and occasionally low observation density. Similarly, poultry and cattle contributions indicated weak spatial patterns (very localized for cattle; relatively uniform for poultry; Fig 4D–4E) with large uncertainties despite smaller nugget-sill ratios.

## Discussion

### Local variation of household welfare indicators masked large-scale patterns

Differences between households at short distance were huge across Uganda. This was indicated by the overall low explanatory power of the regression models using environmental explanatory variables (Tables 3 and 4) and the large nuggets in the variogram models of the regression residuals (Table 5). Spatial predictability using environmental explanatory variables was strongest for highland banana and sorghum contributions, while for most indicators the large local variation masked large-scale patterns, which was apparent both in the regression models and in the residuals (questions I and III). Household resource variables only added explanatory power for the indicators of livestock and cattle contributions (question II).

The spatial patterns of FA matched observations from other studies that identified patterns of food security and poverty across Uganda [7, 8, 29, 40]. However, our results revealed that local variation in FA is much larger than the variability across agroecologies. In a study across East and West Africa, [6] similarly found large local differences in FA within locations in contrast to more gradual differences among locations. Their analysis revealed that household resource characteristics such as farm size overrule agroecological characteristics in determining FA. Although land size was not the most important household variable in our analysis, household resource characteristics (particularly herd size and household size) explained part of the variation in FA. The remaining unexplained (most likely local) variation in the household data could be related to other household characteristics affecting food security, for example education level and age of the household head, social capital (e.g. being part of knowledge networks) or access to market information [41–43], which we did not consider in the analysis.

Key crops for which temperature and rainfall ranges seem to predominantly determine their distribution (highland banana, sorghum) could be linked to FA patterns, while other crops (maize, cassava, beans) were more ubiquitously present across diverse agroecological zones. Sorghum was predominant in the farming systems of the northeast of Uganda, while highland banana was important in the central, west and southwest of the country and largely absent in the north, resembling patterns observed in the past [44]. Our observations match findings by [29] on regional differences of major crops contributing to FA, while our results also show that for all crops the local variation was large.

Similar conclusions can be drawn for livestock, cattle and poultry contributions. Despite the existence of the ‘cattle corridor’ in Uganda [13, 14] and the known association of livestock keeping (particularly poultry production) to urban centres [45], such patterns were not or poorly observed in the livestock, cattle and poultry contributions. The overruling local variation in livestock contribution may have several reasons. One could be that the indicator does not capture all the different contributions of livestock to a Ugandan rural household. In mixed crop-livestock systems in sub-Saharan Africa livestock serves as draught power, provides manure to the crops, is a regular source of food and income to the households and functions as insurance in times of shocks [46]. Income from services such as draught power and manure was not captured in the livestock contribution to FA. Another reason could be that livestock data collection using surveys in sub-Saharan Africa is complicated and data are often unreliable given that these surveys are based on long recall periods, while the farmers usually lack records and are reluctant to share information on wealth indicators like livestock [47]. Lastly, although the number of cattle may be higher in the cattle corridor in Uganda, these consist mainly of large herds that have numerous cattle but few owners. As such, the region may have many cattle, but the majority of its households may not be strongly engaged in cattle keeping themselves or depend on it for their food security.

Similarly, local differences in off-farm income contribution to FA were stronger than large-scale patterns and this may be explained by the diverse ways in which they take shape. Off-farm income sources can be of diverse types. Salary-based off-farm income may be more important in peri-urban areas or in areas with more economic activities [48] and for people of higher education. By contrast, informal off-farm income and remittances may be spread diffusely across the country resulting in weak spatial patterns. For example, migration of household members for off-farm jobs is important for the income (as remittances) of Ugandan households [49].

### Five reasons explain large local variation of regression residuals

Besides above-mentioned *non-spatial variation (I)* at the household level (for example due to education, age or access to information), four additional reasons can explain the large local variation (nugget) that was identified in the regression residuals of the indicators: *II) Missing explanatory variables*: We used a limited set of environmental explanatory variables, while there may be more spatial characteristics that explain variation in FA and the contributing livelihood activities, for example market dynamics or regional governmental programmes promoting agricultural commodities. Additional environmental explanatory variables could contribute to improving the regression model performance and hence reducing the nugget and sill of the regression residual variogram. *III) Spatial mismatch*: The exact locations of the household data were unknown and the environmental explanatory variables that were sampled per household were based on randomly off-set, clustered household locations, which grouped several households on short distances that in reality might live kilometres apart. The clustering of households and the sampling of environmental explanatory variables at the offset locations introduced noise, which affected both the performance of the regression models using environmental explanatory variables and the nugget of the variogram models. Performing the analyses on the real locations of the households (and their fields) is expected to reduce the level of local variation and improve information on large-scale patterns in response to environmental explanatory variables. *IV) Measurement errors*: Information on FA and livelihood activities was obtained from survey data, known to be subject to constraints and erroneous information [47, 50, 51]. *V) Model structural error*: The model used functions to approximate trends, while these may not reflect real structures. For example, using linear models on non-linear effects might have introduced structural error. Sophisticated predictive techniques such as machine learning [5] are compatible with our procedure and could improve the regression predictions.

### Interventions must recognise the diversity in livelihood activities within areas

Our procedure enables to systematically evaluate spatial patterns and the quality of maps of farming systems and household welfare and adds to existing approaches in which local variation often remains hidden [7]. The latter can be misleading if only larger patterns are shown without indicating how much of the total variation is explained by these patterns. Our results reveal that local differences in welfare and welfare-related activities can be large, which has implications for the planning of interventions. For example, our findings on the large local variation in livestock contribution to FA indicate that in Uganda’s cattle corridor as well as in other regions there is a large local diversity in livestock ownership, which needs to be considered in targeting livestock interventions. Our cattle map shows that dependency of the households on cattle for income and food security in the cattle corridor is not substantially different to other areas in Uganda. Earlier work has shown that small livestock was more important in contributing to food availability for the poorer households, whereas cattle was important for the wealthier households [9]. When targeting the poor, interventions focusing on small livestock therefore remain relevant, also within cattle areas. Similarly, despite revealing distribution patterns that resemble those of other existing maps, the contributions of major crops contain large local variation. For example, within Uganda’s banana-coffee system areas, smallholders exist that have little or no banana or coffee in their system. Interventions that aim to target the poor must thus recognise that diversity in livelihood activities for income generation within any given area often overrides the variability of livelihood activities between distant regions in the country.

## Conclusions

We applied a stepwise procedure to 1927 farm households across Uganda to identify country-wide patterns of indicators on FA and contributing livelihood activities using spatially explicit environmental and socio-economic data and household resource characteristics as explanatory variables. With few exceptions, predictions of the indicators were weak, highlighting the difficulty in capturing variability at larger scale. Also household explanatory variables identified little additional variation compared to environmental explanatory variables alone. Spatial predictability was strongest for indicators for which environmental gradients determined their distribution, such as highland banana contribution to the crop part of FA. In contrast, indicators of crops that were more ubiquitously present across agroecological zones showed large local variation, which often overruled large-scale patterns (e.g. cassava and maize contributions to the crop part of FA, and livestock and off-farm income contributions to FA).

Our procedure enables to systematically evaluate spatial patterns of farming systems and household welfare (e.g. food security) and to quantify local and large-scale variation. Thereby, it adds to existing approaches, which often only address large-scale patterns and, given the substantial local variation observed, may hide relevant heterogeneity. This has implications for planning of interventions. Decision makers targeting interventions in an area such as the Ugandan cattle corridor need to take into account that the importance of cattle for the livelihoods of the households in these areas varies enormously. While the cattle corridor may harbour many cattle, these belong to few herd owners. Instead, for targeting the poor, interventions on small livestock may be more relevant. Similarly, the importance of crops such as banana or coffee for household welfare in areas where banana-coffee systems are predominant varies largely. Interventions that aim to target the poor must thus recognise the large diversity in livelihood activities within any given area, which often overrides the variability between regions. Our approach generates spatially continuous and quantitative information on livelihood activities for food availability, including a quantification of uncertainty in these patterns, and provides a basis for further analyses to identify vulnerability of different regions and households to future changes by linking this approach to scenarios on climate change and price variability.

## Supporting information

S1 Fig Maps of key indicators using LSMS data from 2010/11 and 2011/12. Overall patterns of the maps of key indicators for the LSMS data from 2010/11 and 2011/12 were similar with largest differences for food availability (FA) and the cattle contribution.
(PDF)

S2 Fig Difference maps comparing LSMS data from 2010/11 and 2011/12. Maps from 2011/12 are subtracted from maps from 2010/11. Positive results (green, yellow) indicate that FA or the contribution of the variable in 2010/11 was larger than in 2011/12. Negative results (white) indicate that FA or the contribution of the variable in 2010/11 was smaller than in 2011/12. FA, cassava contribution (some regions) and cattle contribution tended to be larger in 2010/11 than in 2011/12.
(PDF)

S3 Fig Root mean squared error maps comparing LSMS data from 2010/11 and 2011/12. Root mean squared error was calculated as: √((*LSMS*201011 − *LSMS*20112)^2^). It gives an indication about the spread between the two years. Maps indicate that differences between the two years were locally large (green) for banana and cattle contributions and less for cassava contribution and FA.
(PDF)

S4 Fig Variogram models of the indicators. (PDF)

S1 Table Food availability and livelihood activities as dependent variables for the regression analyses. (PDF)

S2 Table Regression coefficients and significance of environmental explanatory variables in multiple inflated beta regression model (MIBR-EV) for the dependent variables ‘crops’, ‘livestock’ and ‘off-farm income’ as livelihood activities contributing to food availability. (PDF)

S3 Table Regression coefficients and significance of environmental explanatory variables multiple inflated beta regression model (MIBR-EV) for major crops contributing to the livelihood activity ‘crops’. (PDF)

S4 Table Regression coefficients and significance of environmental explanatory variables multiple inflated beta regression model (MIBR-EV) for major livestock groups ‘cattle’ and ‘poultry’ contributing to the livelihood activity ‘livestock’. (PDF)

S5 Table Regression coefficients and significance of environmental and household level explanatory variables in multiple inflated beta regression model (MIBR-EVHR) for the dependent variables ‘crops’, ‘livestock’ and ‘off-farm income’ as livelihood activities contributing to food availability. (PDF)

S6 Table Regression coefficients and significance of environmental and household level explanatory variables multiple inflated beta regression model (MIBR-EVHR) for major crops contributing to the livelihood activity ‘crops’. (PDF)

S7 Table Regression coefficients and significance of environmental and household level explanatory variables multiple inflated beta regression model (MIBR-EVHR) for major livestock groups contributing to the livelihood activity ‘livestock’. (PDF)
