# Supplementary material for: Using household survey data to identify large-scale food security patterns across Uganda
Source: PLoS One. 2018 Dec 13;13(12):e0208714. doi: 10.1371/journal.pone.0208714 (PMC6292625; doi:10.1371/journal.pone.0208714)

LSMS 2010-11 **LSMS 2011-12**

Food availability (kcal cap^{-1} d^{-1})

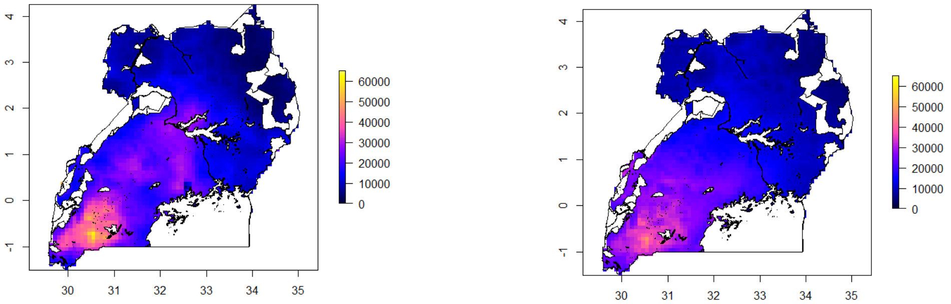

Banana contribution

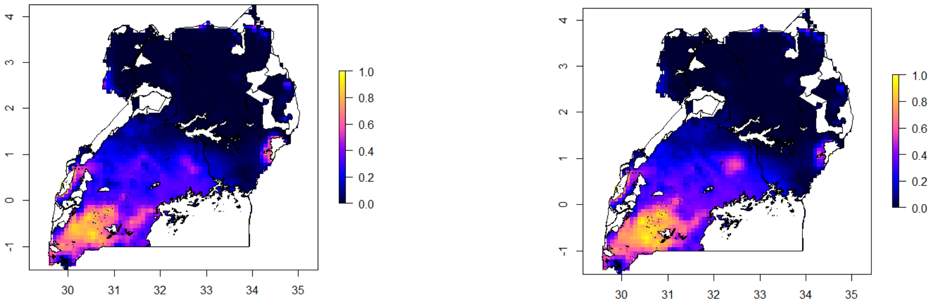

Cassava contribution

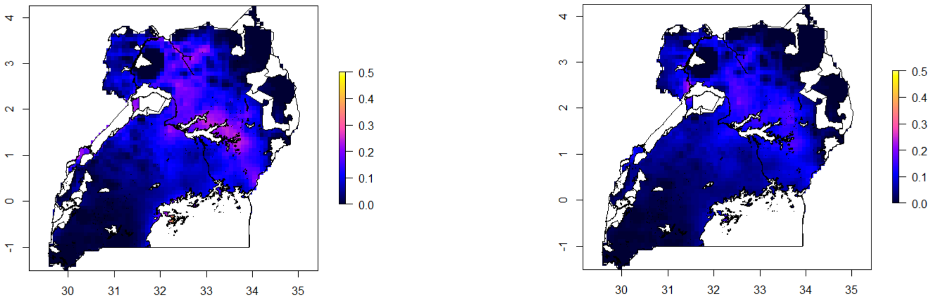

Cattle contribution

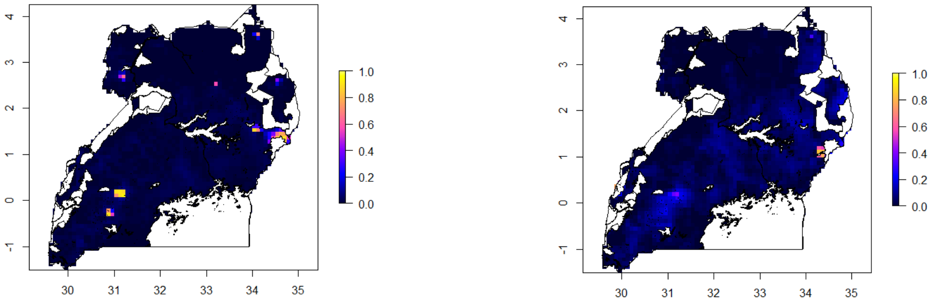

Supplement: S1 Fig — Overall patterns of the maps of key indicators for the LSMS data from 2010/11 and 2011/12 were similar with largest differences for food availability (FA) and the cattle contribution. (PDF) [file pone.0208714.s001.pdf]
