# Supplementary figures and images for: Using household survey data to identify large-scale food security patterns across Uganda
Source: PLoS One. 2018 Dec 13;13(12):e0208714. doi: 10.1371/journal.pone.0208714 (PMC6292625; doi:10.1371/journal.pone.0208714)

**banana difference (2010/11-2011/12)**

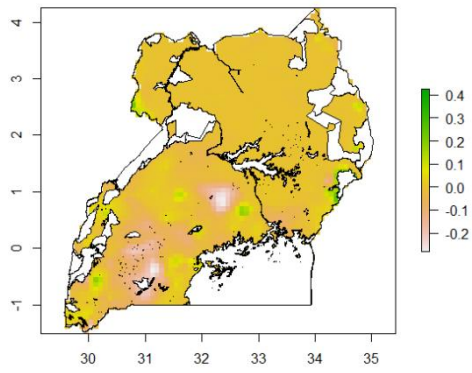

**cassava difference (2010/11-2011/12)**

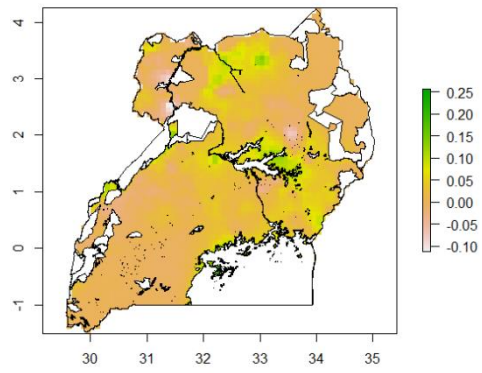

**cattle difference (2010/11-2011/12)**

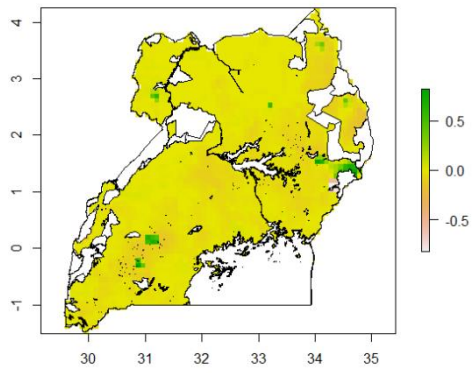

**FA difference (2010/11-2011/12)**

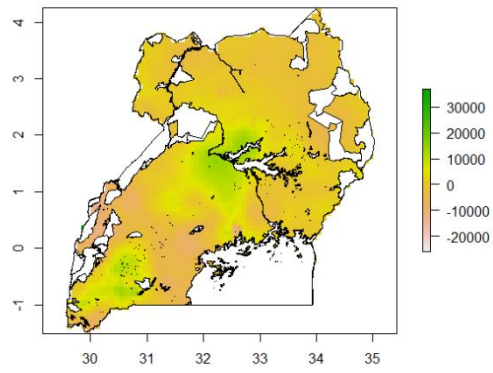

Supplement: S2 Fig — Maps from 2011/12 are subtracted from maps from 2010/11. Positive results (green, yellow) indicate that FA or the contribution of the variable in 2010/11 was larger than in 2011/12. Negative results (white) indicate that FA or the contribution of the variable in 2010/11 was smaller than in 2011/12. FA, cassava contribution (some regions) and cattle contribution tended to be larger in 2010/11 than in 2011/12. (PDF) [file pone.0208714.s002.pdf]

**banana rmse (2010/11-2011/12)**

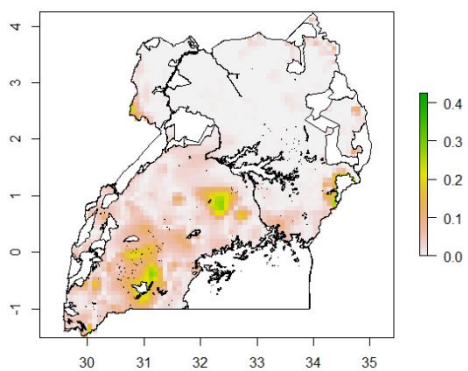

**cassava rmse (2010/11-2011/12)**

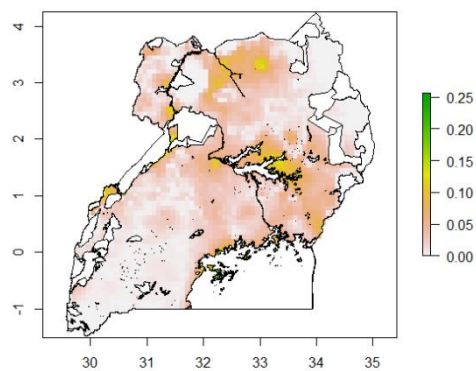

**cattle rmse (2010/11-2011/12)**

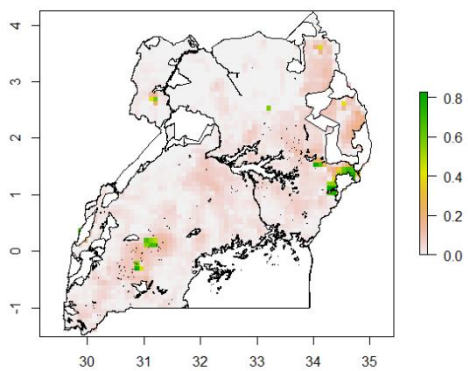

**FA rmse (2010/11-2011/12)**

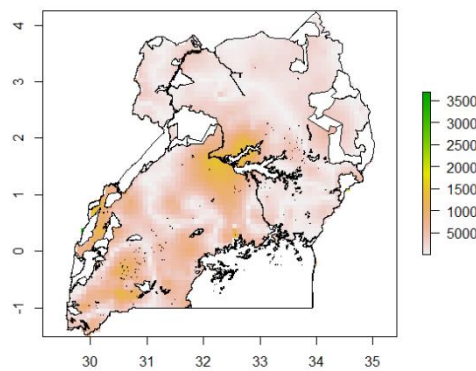

Supplement: S3 Fig — Root mean squared error was calculated as: √((LSMS201011 − LSMS20112)2). It gives an indication about the spread between the two years. Maps indicate that differences between the two years were locally large (green) for banana and cattle contributions and less for cassava contribution and FA. (PDF) [file pone.0208714.s003.pdf]

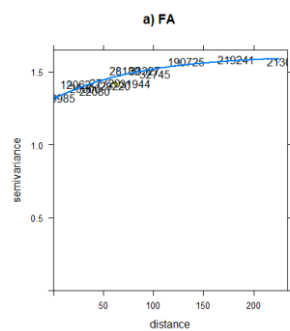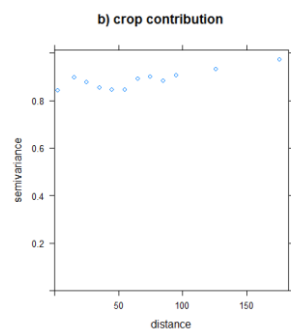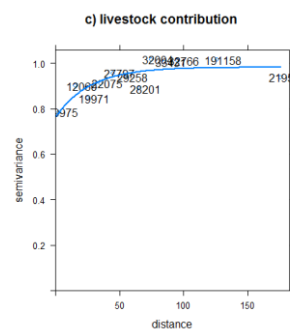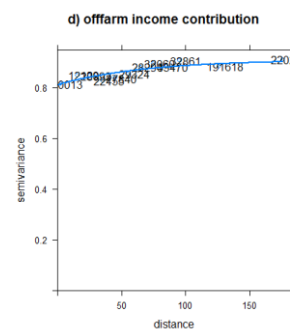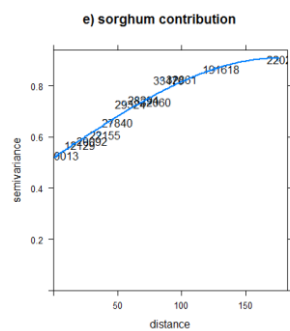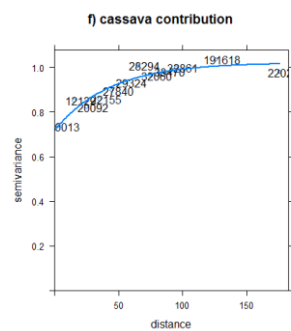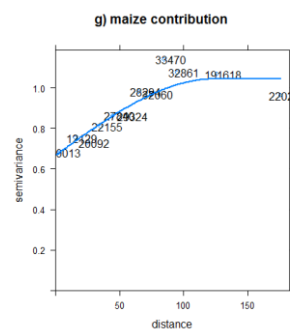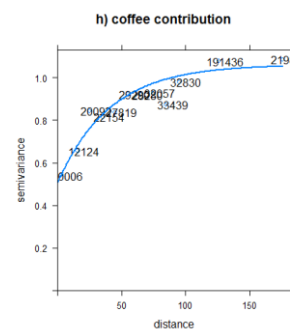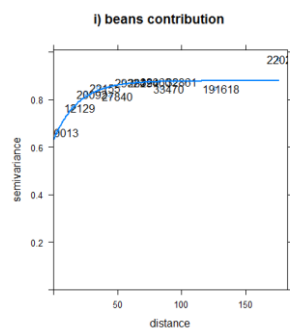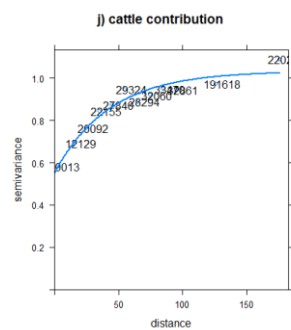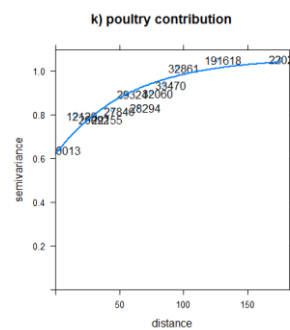

Supplement: S4 Fig — (PDF) [file pone.0208714.s004.pdf]
