# Supplementary material for: Using household survey data to identify large-scale food security patterns across Uganda
Source: PLoS One. 2018 Dec 13;13(12):e0208714. doi: 10.1371/journal.pone.0208714 (PMC6292625; doi:10.1371/journal.pone.0208714)
Supplement: S1 Table — (PDF) [file pone.0208714.s005.pdf]

**Dependent variable**	**min**	**1st qu.**	**median**	**mean**	**3rd qu.**	**max**	**Regression model***
Food Availability (FA)	5	3 x 10^3	6 x 10^3	20x 10^3	14x 10^3	93x10^5	MLR
Livelihood activities contributing to FA							
Crops	0	0.31	0.78	0.65	1.0	1.0	MIBR
Livestock	0	0	0	0.09	0.08	1.0	MIBR-0
Off-farm income	0	0	0	0.26	0.51	1.0	MIBR
Crops contributing to the livelihood activity 'crops'							
Banana	0	0	0.03	0.26	0.54	1.0	MIBR
Sorghum	0	0	0	0.05	0	1.0	MIBR
Cassava	0	0	0.01	0.11	0.13	1.0	MIBR
Maize	0	0	0.06	0.16	0.24	1.0	MIBR
Coffee	0	0	0	0.05	0	1.0	MIBR-0
Beans	0	0	0.04	0.10	0.14	1.0	MIBR
Livestock types contributing to the livelihood activity 'livestock'							
Cattle	0	0	0	0.16	0	1.0	MIBR
Poultry	0	0	0	0.09	0	1.0	MIBR

*MLR: Multiple linear regression model; MIBR: Multiple zero-and-one inflated beta regression model; MIBR-0: Multiple zero inflated beta regression model
