# Supplementary material for: Using household survey data to identify large-scale food security patterns across Uganda
Source: PLoS One. 2018 Dec 13;13(12):e0208714. doi: 10.1371/journal.pone.0208714 (PMC6292625; doi:10.1371/journal.pone.0208714)
Supplement: S2 Table — (PDF) [file pone.0208714.s006.pdf]

Parameter		Crops	Livestock	Off-farm income
μ_1	DEM	-	-	-6.2x 10^{-4}***
μ_2	TEMP	-	-	-
μ_3	TEMP_R	-	-	-7.7 x 10^{-3}*
μ_4	PREC	-	-	-
μ_5	PREC_S	-1.9x 10^{-2}**	-	-
μ_6	LGP	6.6x 10^{-3}***	-5.6x 10^{-3}**	-4.5x 10^{-3}*
μ_7	SCARB	-	-	-
μ_8	POP	-	2.6x 10^{-2}**	4.3x 10^{-2}**
μ_9	TRAV	-	8.2x 10^{-4}.	1.2x 10^{-3}*
σ_1	DEM	-	-	-4.4x 10^{-4}***
σ_2	TEMP	-	-	-
σ_3	TEMP_R	-	-	6.5x 10^{-3}**
σ_4	PREC	-	-	-
σ_5	PREC_S		-	-
σ_6	LGP	3.8x 10^{-3}***	4.5x 10^{-3}*	-
σ_7	SCARB	-	-	-
σ_8	POP	-	-	-
σ_9	TRAV	-	-	1.2x 10^{-3}***
ν_1	DEM	-	-	-
ν_2	TEMP	-	-	-
ν_3	TEMP_R	-	-	-
ν_4	PREC	-	-	-
ν_5	PREC_S	1.1x 10^{-1}***	-	-
ν_6	LGP	-	-	5.7x 10^{-3}**
ν_7	SCARB	-	-	-
ν_8	POP	-	3.9x 10^{-2}**	-
ν_9	TRAV	-	3.1x 10^{-3}***	-
τ_1	DEM	-	*n.a.*	-
τ_2	TEMP	-	*n.a.*	-
τ_3	TEMP_R	-	*n.a.*	-
τ_4	PREC	-	*n.a.*	-
τ_5	PREC_S	-	*n.a.*	-
τ_6	LGP	-	*n.a.*	-3.3x 10^{-2}**
τ_7	SCARB	-	*n.a.*	-
τ_8	POP	-	*n.a.*	-
τ_9	TRAV	1.4x 10^{-3}*	*n.a.*	-
Pseudo R^2		0.02	0.003	0.01
AIC_{ini} - AIC_{final}		77	47	91

Significance: *** < 0.001, ** < 0.01, * < 0.05, **.** <0.1

For explanation of model parameter see Material and Methods. Environmental explanatory variables: DEM = elevation, TEMP = average annual mean temperature, TEMP_R = average annual temperature range, PREC = average annual precipitation, PREC_S = average annual precipitation variation, LGP =

average length of growing period, SCARB = soil carbon stock, POP = human population density, TRAV = market access in travel time to nearest town of +50,000 inhabitants
