# Supplementary material for: Using household survey data to identify large-scale food security patterns across Uganda
Source: PLoS One. 2018 Dec 13;13(12):e0208714. doi: 10.1371/journal.pone.0208714 (PMC6292625; doi:10.1371/journal.pone.0208714)
Supplement: S3 Table — (PDF) [file pone.0208714.s007.pdf]

Parameter		Banana	Sorghum	Maize	Cassava	Coffee	Beans
μ_1	DEM	-	-	-	-	-	-
μ_2	TEMP	-3.3x 10^{-2}***	-	1.0x 10^{-2}***	4.6x 10^{-2}***	2.0x 10^{-2}***	-
μ_3	TEMP_R	-7.8x 10^{-3}.	-1.9x 10^{-2}***	-	-	-	-
μ_4	PREC	-1.1x 10^{-3}***	-	-	2.0x 10^{-3}***	-	-
μ_5	PREC_S	-	5.3x 10^{-2}***	-	-	-	-
μ_6	LGP	-	-2.0x 10^{-2}***	-2.6x 10^{-3}.	-	9.9x 10^{-3}**	-
μ_7	SCARB	1.1x 10^{-3}	-	-	-	-	8.8x 10^{-6}***
μ_8	POP	-	-	-	-	-	-
μ_9	TRAV	-	-	-	-	-	-
σ_1	DEM	-	-	-	-	-	-
σ_2	TEMP	-	-	-3.5x 10^{-3}*	2.6x 10^{-2}***	-1.4x 10^{-2}**	-
σ_3	TEMP_R	-	-	-	-	-	-
σ_4	PREC	-	-	-	1.1x 10^{-3}***	-	-
σ_5	PREC_S	-	-	-	-	-	-
σ_6	LGP	-	-5.7x 10^{-3}**	-4.6x 10^{-3}***	-	-	-
σ_7	SCARB	-	-	-	-	-	-
σ_8	POP	-	-	-	-	-	-
σ_9	TRAV	-	-	-	-	-	-
ν_1	DEM	-	-	-	-	-	-
ν_2	TEMP	2.5x 10^{-2}***	-	-	-3.6x 10^{-2}***	2.4x 10^{-2}***	5.0x 10^{-2}***
ν_3	TEMP_R	8.9x 10^{-2}***	2.5x 10^{-2}***	9.0x 10^{-3}.	1.8x 10^{-2}**	-	-
ν_4	PREC	-7.7x 10^{-4}*	-	-	-1.5x 10^{-3}***	-	-
ν_5	PREC_S	-	-1.0x 10^{-3}***	-	-	-	-
ν_6	LGP	-	3.0x 10^{-2}***	-1.5x 10^{-2}***	-1.2x 10^{-2}***	-1.8x 10^{-2}***	-
ν_7	SCARB	-4.8x 10^{-5}***	-	3.7x 10^{-5}***	-	-	-4.4x 10^{-5}***
ν_8	POP	-	-	-	-	-	-
ν_9	TRAV	3.3x 10^{-3}***	-	-	3.5x 10^{-3}***	3.2x 10^{-3}***	-
τ_1	DEM	-	-	-	-	*n.a.*	-
τ_2	TEMP	-	-	-	5.1x 10^{-2}*	*n.a.*	-
τ_3	TEMP_R	-	-	8.7 x 10^{-2}***	-	*n.a.*	-
τ_4	PREC	-	-	-	-	*n.a.*	-
τ_5	PREC_S	-	-	-	-	*n.a.*	-
τ_6	LGP	-	-5.1x 10^{-2}***	-3.5x 10^{-2}**	-	*n.a.*	-
τ_7	SCARB	-	-1.0x 10^{-4}*	7.5x 10^{-5}*	-	*n.a.*	-
τ_8	POP	-	-	-	-	*n.a.*	-
τ_9	TRAV	-	-	-	-	*n.a.*	-
Pseudo R^2		0.35	0.53	0.02	0.12	0.05	0.04
AIC_{ini} - AIC_{final}		1052	530	141	573	197	390

Significance: *** < 0.001, ** < 0.01, * < 0.05, **.** < 0.1

For explanation of model parameter see Material and Methods. Environmental explanatory variables: DEM = elevation, TEMP = average annual mean temperature, TEMP_R = average annual temperature range, PREC = average annual precipitation, PREC_S = average annual precipitation variation, LGP =

average length of growing period, SCARB = soil carbon stock, POP = human population density, TRAV = market access in travel time to nearest town of +50,000 inhabitants.
