# Supplementary material for: Using household survey data to identify large-scale food security patterns across Uganda
Source: PLoS One. 2018 Dec 13;13(12):e0208714. doi: 10.1371/journal.pone.0208714 (PMC6292625; doi:10.1371/journal.pone.0208714)
Supplement: S4 Table — (PDF) [file pone.0208714.s008.pdf]

Parameter	Cattle	Poultry
μ_1 DEM	-	-
μ_2 TEMP	-	-
μ_3 TEMP_R	-	-
μ_4 PREC	-	-
μ_5 PREC_S	-	-
μ_6 LGP	-	-
μ_7 SCARB	-	-
μ_8 POP	-	-
μ_9 TRAV	-	-
σ_1 DEM	-	-
σ_2 TEMP	-	-
σ_3 TEMP_R	-	-
σ_4 PREC	-	-
σ_5 PREC_S	-	2.2x 10^{-2}.
σ_6 LGP	-	-
σ_7 SCARB	-	-
σ_8 POP	-	-
σ_9 TRAV	-2.3x 10^{-3}**	-
ν_1 DEM	-	-
ν_2 TEMP	-	-
ν_3 TEMP_R	-	-
ν_4 PREC	-	-
ν_5 PREC_S	-	-4.2x 10^{-2}***
ν_6 LGP	-	-
ν_7 SCARB	-	-
ν_8 POP	-	-
ν_9 TRAV	1.7x 10^{-3}**	5.3x 10^{-3}***
τ_1 DEM	-	-
τ_2 TEMP	-	-
τ_3 TEMP_R	-	-
τ_4 PREC	-	-
τ_5 PREC_S	-3.1x 10^{-2}**	-
τ_6 LGP	-	-
τ_7 SCARB	-	-
τ_8 POP	-	-
τ_9 TRAV	-	-
Pseudo R^2	0.005	0.01
AIC_{ini} - AIC_{final}	24	40

Significance: *** < 0.001, ** < 0.01, * < 0.05, **.** < 0.1

For explanation of model parameter see Material and Methods. Environmental explanatory variables: DEM = elevation, TEMP = average annual mean temperature, TEMP_R = average annual temperature

range, PREC = average annual precipitation, PREC_S = average annual precipitation variation, LGP = average length of growing period, SCARB = soil carbon stock, POP = human population density, TRAV = market access in travel time to nearest town of +50,000 inhabitants.
