# Supplementary material for: Using household survey data to identify large-scale food security patterns across Uganda
Source: PLoS One. 2018 Dec 13;13(12):e0208714. doi: 10.1371/journal.pone.0208714 (PMC6292625; doi:10.1371/journal.pone.0208714)
Supplement: S5 Table — (PDF) [file pone.0208714.s009.pdf]

Parameter		Crops	Livestock	Off-farm income
μ_1	DEM	-	-	-x 10^{-4}***
μ_2	TEMP	-	-	-
μ_3	TEMP_R	-	-	-
μ_4	PREC	-	-	-
μ_5	PREC_S	-2.3x 10^{-2}***	-	-
μ_6	LGP	5.7x 10^{-3}**	-7.2x 10^{-3}***	-2.7x 10^{-3}
μ_7	SCARB	-	-	-
μ_8	POP	-	2.8x 10^{-2}**	4.4x 10^{-2}***
μ_9	TRAV	-	-	1.2x 10^{-3}*
μ_{10}	TLU	-4.3x 10^{-2}***	3.6x 10^{-2}***	-4.4x 10^{-2}***
μ_{11}	HH_SIZE	-4.2x 10^{-2}***	-	3.4x 10^{-2}**
μ_{12}	LAND	7.8x 10^{-3}*	-	-8.4x 10^{-3}**
σ_1	DEM	-	-	-4.6x 10^{-4}***
σ_2	TEMP	-	-	-
σ_3	TEMP_R	-	-	6.7x 10^{-3}**
σ_4	PREC	-	-	-
σ_5	PREC_S	-	-	-
σ_6	LGP	3.7x 10^{-3}***	7.4x 10^{-3}***	-
σ_7	SCARB	-	-	-
σ_8	POP	-	-	-
σ_9	TRAV	-	-	1.2x 10^{-3}***
σ_{10}	TLU	-3.9x 10^{-2}***	-1.6x 10^{-2}*	
σ_{11}	HH_SIZE	-1.7x 10^{-2}*	-	
σ_{12}	LAND	-	-	-3.1x 10^{-2}***
ν_1	DEM	-	-	-
ν_2	TEMP	-	-	-
ν_3	TEMP_R	-	-	-
ν_4	PREC	-	-	-
ν_5	PREC_S	1.2x 10^{-1}***	-	-
ν_6	LGP	-	-	6.1x 10^{-3}**
ν_7	SCARB	-	-	-
ν_8	POP	-	1.9x 10^{-2}.	-
ν_9	TRAV	-	3.5x 10^{-3}***	-
ν_{10}	TLU	4.0x 10^{-2}.	-4.4x 10^{-1}***	-
ν_{11}	HH_SIZE	-	-4.6x 10^{-2}**	-5.4x 10^{-2}***
ν_{12}	LAND	-	-	-
τ_1	DEM	-	*n.a.*	-
τ_2	TEMP	-	*n.a.*	-
τ_3	TEMP_R	-	*n.a.*	-
τ_4	PREC	-	*n.a.*	-
τ_5	PREC_S	-	*n.a.*	-
τ_6	LGP	-	*n.a.*	-3.3x 10^{-2}**
τ_7	SCARB	-	*n.a.*	-

τ_8	POP	-	*n.a.*	-
τ_9	TRAV	1.2x 10^{-3}*	*n.a.*	-
τ_{10}	TLU	-2.3x 10^{-1}***	*n.a.*	-
τ_{11}	HH_SIZE	-6.0x 10^{-2}**	*n.a.*	-
τ_{12}	LAND	-	*n.a.*	-
Pseudo R^2		0.05	0.17	0.02
AIC_{ini} - AIC_{final}		291	443	134

Significance: *** < 0.001, ** < 0.01, * < 0.05, **.** < 0.1

For explanation of model parameter see Material and Methods. Environmental explanatory variables: DEM = elevation, TEMP = average annual mean temperature, TEMP_R = average annual temperature range, PREC = average annual precipitation, PREC_S = average annual precipitation variation, LGP = average length of growing period, SCARB = soil carbon stock, POP = human population density, TRAV = market access in travel time to nearest town of +50,000 inhabitants, TLU = tropical livestock unit, HH_SIZE = number of household members, LAND = total cultivated land area.
