# Supplementary material for: Using household survey data to identify large-scale food security patterns across Uganda
Source: PLoS One. 2018 Dec 13;13(12):e0208714. doi: 10.1371/journal.pone.0208714 (PMC6292625; doi:10.1371/journal.pone.0208714)
Supplement: S6 Table — (PDF) [file pone.0208714.s010.pdf]

Parameter		Banana	Sorghum	Maize	Cassava	Coffee	Beans
μ_1	DEM	-	-	-	-	-	-
μ_2	TEMP	-	-	$1.1x\ 10^{-2}$***	$4.6x\ 10^{-2}$***	$1.9x\ 10^{-2}$***	-
μ_3	TEMP_R	-	$-1.9x\ 10^{-2}$***	-	-	-	-
μ_4	PREC	-	-	-	$2.0x\ 10^{-3}$***	-	-
μ_5	PREC_S	-	$5.1x\ 10^{-2}$***	-	-	-	-
μ_6	LGP	-	$-2.0x\ 10^{-2}$***	-	-	$9.5x\ 10^{-3}$**	-
μ_7	SCARB	-	-	-	-	-	$7.8x\ 10^{-6}$**
μ_8	POP	-	-	-	-	-	-
μ_9	TRAV	-	-	-	-	-	-
μ_{10}	TLU	-	-	-	-	-	-
μ_{11}	HH_SIZE	-	-	$-3.2x\ 10^{-2}$***	$-2.6x\ 10^{-2}$**	-	$-4.0x\ 10^{-2}$***
μ_{12}	LAND	-	-	-	-	-	-
σ_1	DEM	-	-	-	-	-	-
σ_2	TEMP	-	-	$-3.9x\ 10^{-3}$*	$2.6x\ 10^{-2}$***	$-1.5x\ 10^{-2}$**	-
σ_3	TEMP_R	-	-	-	-	-	-
σ_4	PREC	-	-	-	$1.2x\ 10^{-3}$***	-	-
σ_5	PREC_S	-	-	-	-	-	-
σ_6	LGP	-	$-5.9x\ 10^{-3}$**	$-4.5x\ 10^{-3}$***	-	-	-
σ_7	SCARB	-	-	-	-	-	-
σ_8	POP	-	-	-	-	-	-
σ_9	TRAV	-	-	-	-	-	-
σ_{10}	TLU	-	$-2.9x\ 10^{-2}$.	-	-	$-5.0x\ 10^{-2}$*	-
σ_{11}	HH_SIZE	-	-	$-1.9x\ 10^{-2}$*	-	-	$-2.2x\ 10^{-2}$**
σ_{12}	LAND	-	-	-	-	-	-
ν_1	DEM	-	-	-	-	-	-
ν_2	TEMP	-	-	-	$-3.6x\ 10^{-2}$***	-	$5.0x\ 10^{-2}$***
ν_3	TEMP_R	-	$2.5x\ 10^{-2}$***	-	$1.8x\ 10^{-2}$**	$2.4x\ 10^{-2}$***	-
ν_4	PREC	-	-	-	$-1.5x\ 10^{-3}$***	-	-
ν_5	PREC_S	-	$-9.9x\ 10^{-2}$***	-	-	-	-
ν_6	LGP	-	$3.0x\ 10^{-2}$***	$-1.6x\ 10^{-2}$***	$-1.2x\ 10^{-2}$***	$-1.8x\ 10^{-2}$***	-
ν_7	SCARB	-	-	$3.1x\ 10^{-5}$***	-	-	$-4.4x\ 10^{-5}$***
ν_8	POP	-	-	-	-	-	-
ν_9	TRAV	-	-	-	$3.5x\ 10^{-3}$***	$3.0x\ 10^{-3}$***	-
ν_{10}	TLU	-	-	-	-	$6.3x\ 10^{-2}$**	-
ν_{11}	HH_SIZE	-	-	$-5.4x\ 10^{-2}$***	-	$-4.2x\ 10^{-2}$*	-
ν_{12}	LAND	-	-	-	-	-	-
τ_1	DEM	-	-	-	-	*n.a.*	-
τ_2	TEMP	-	-	-	$5.9x\ 10^{-2}$**	*n.a.*	-
τ_3	TEMP_R	-	-	$8.3x\ 10^{-2}$***	-	*n.a.*	-
τ_4	PREC	-	-	-	-	*n.a.*	-
τ_5	PREC_S	-	-	-	-	*n.a.*	-
τ_6	LGP	-	$-5.1x\ 10^{-2}$***	$-3.6x\ 10^{-2}$**	-	*n.a.*	-
τ_7	SCARB	-	$-1.1x\ 10^{-4}$*	$7.2x\ 10^{-5}$*	-	*n.a.*	-

τ_8	POP	-	-	-	-	*n.a.*	-
τ_9	TRAV	-	-	-	-	*n.a.*	-
τ_{10}	TLU	-	-5.7x 10^{-1}.	-	-	-	-
τ_{11}	HH_SIZE	-	-	-	-	-	-
τ_{12}	LAND	-	-	-	-7.3x 10^{-1}.	-	-
Pseudo R^2		*Model not*	0.54	0.02	0.12	0.06	0.05
AIC_{ini} - AIC_{final}		*converging*	538	159	585	212	405

Significance: *** < 0.001, ** < 0.01, * < 0.05, **.** < 0.1

For explanation of model parameter see Material and Methods. Environmental explanatory variables: DEM = elevation, TEMP = average annual mean temperature, TEMP_R = average annual temperature range, PREC = average annual precipitation, PREC_S = average annual precipitation variation, LGP = average length of growing period, SCARB = soil carbon stock, POP = human population density, TRAV = market access in travel time to nearest town of +50,000 inhabitants, TLU = tropical livestock unit, HH_SIZE = number of household members, LAND = total cultivated land area.
