# Supplementary material for: Using household survey data to identify large-scale food security patterns across Uganda
Source: PLoS One. 2018 Dec 13;13(12):e0208714. doi: 10.1371/journal.pone.0208714 (PMC6292625; doi:10.1371/journal.pone.0208714)
Supplement: S7 Table — (PDF) [file pone.0208714.s011.pdf]

Parameter		Cattle	Poultry
μ_1	DEM	-	-
μ_2	TEMP	-	-
μ_3	TEMP_R	-	-
μ_4	PREC	-	-
μ_5	PREC_S	-	-
μ_6	LGP	-	-
μ_7	SCARB	-	-
μ_8	POP	-	-
μ_9	TRAV	-	-
μ_{10}	TLU	-	-
μ_{11}	HH_SIZE	-	-
μ_{12}	LAND	-	-
σ_1	DEM	-	-
σ_2	TEMP	-	-
σ_3	TEMP_R	-	-
σ_4	PREC	-	-
σ_5	PREC_S	-	2.2x 10^{-2}.
σ_6	LGP	-	-
σ_7	SCARB	-	-
σ_8	POP	-	-
σ_9	TRAV	-2.3x 10^{-3}**	-
σ_{10}	TLU	-	-
σ_{11}	HH_SIZE	-	-
σ_{12}	LAND	-	-
ν_1	DEM	-	-
ν_2	TEMP	-	-
ν_3	TEMP_R	-	-
ν_4	PREC	-	-
ν_5	PREC_S	-	-4.4x 10^{-2}***
ν_6	LGP	-	-
ν_7	SCARB	-	-
ν_8	POP	-	-
ν_9	TRAV	3.1x 10^{-3}***	5.5x 10^{-3}***
ν_{10}	TLU	-5.4x 10^{-1}***	-5.0x 10^{-2}***
ν_{11}	HH_SIZE	-9.5x 10^{-2}***	-
ν_{12}	LAND	-	-
τ_1	DEM	-	-
τ_2	TEMP	-	-
τ_3	TEMP_R	-	-
τ_4	PREC	-	-
τ_5	PREC_S	-5.6x 10^{-2}***	-
τ_6	LGP	-	-
τ_7	SCARB	-	-

τ_8	POP	-	-
τ_9	TRAV	-	-
τ_{10}	TLU	-	$-1.8x\ 10^{-1}$***
τ_{11}	HH_SIZE	$-5.4x\ 10^{-2}$*	-
τ_{12}	LAND	-	-
Pseudo R^2		0.29	0.007
AIC_{ini} - AIC_{final}		535	63

Significance: *** < 0.001, ** < 0.01, * < 0.05, **.** < 0.1

For explanation of model parameter see Material and Methods. Environmental explanatory variables: DEM = elevation, TEMP = average annual mean temperature, TEMP_R = average annual temperature range, PREC = average annual precipitation, PREC_S = average annual precipitation variation, LGP = average length of growing period, SCARB = soil carbon stock, POP = human population density, TRAV = market access in travel time to nearest town of +50,000 inhabitants, TLU = tropical livestock unit, HH_SIZE = number of household members, LAND = total cultivated land area.
